# Electrochemical sensors and devices for heavy metals assay in water: the French groups' contribution

**DOI:** 10.3389/fchem.2014.00019

**Published:** 2014-04-30

**Authors:** Luca Pujol, David Evrard, Karine Groenen-Serrano, Mathilde Freyssinier, Audrey Ruffien-Cizsak, Pierre Gros

**Affiliations:** ^1^Université de Toulouse, UPS, INPT, Laboratoire de Génie ChimiqueToulouse, France; ^2^CNRS, Laboratoire de Génie ChimiqueToulouse, France; ^3^EltaBlagnac, France

**Keywords:** electrochemical detection, heavy metals, carbon electrode, polarography, mercury-free electrode, chemically modified electrode, ion selective electrode, speciation

## Abstract

A great challenge in the area of heavy metal trace detection is the development of electrochemical techniques and devices which are user-friendly, robust, selective, with low detection limits and allowing fast analyses. This review presents the major contribution of the French scientific academic community in the field of electrochemical sensors and electroanalytical methods within the last 20 years. From the well-known polarography to the up-to-date generation of functionalized interfaces, the different strategies dedicated to analytical performances improvement are exposed: stripping voltammetry, solid mercury-free electrode, ion selective sensor, carbon based materials, chemically modified electrodes, nano-structured surfaces. The paper particularly emphasizes their advantages and limits face to the last Water Frame Directive devoted to the Environmental Quality Standards for heavy metals. Recent trends on trace metal speciation as well as on automatic “on line” monitoring devices are also evoked.

## Introduction

Like many other micropollutants such as drugs or cosmetics and their by-products, pesticides and industrial or household chemicals, heavy metals represent a growing environmental (Callender, 2004; Roy, 2010) and health (Musarrat et al., 2011; Prabhakar et al., 2012) problem. They may be considered as a major source of ecological issues due to their wide overspread in natural media (Mhatre, 1995). Although naturally produced throughout biogeochemical processes, heavy metals occurrence in the environment mainly originates from human activities: air emissions from coal-burning plants, smelters, waste incinerators, process wastes from mining, industrial and urban runoff all participate to their wide spreading (Friedman et al., 1993; Lindqvist, 1995; Nagajyoti et al., 2010). Once released to the environment, these metals can remain for decades or centuries since they are not biodegradable. Depending on the contamination pathway, they appear at detectable levels in food resources such as vegetables, grains or fruits, and fish or shellfish throughout bioaccumulation all along the trophic chain, thus contaminating the final consumer—human being (Musarrat et al., 2011; Prabhakar et al., 2012). Another contamination way is direct intoxication from domestic environment, for instance lead traces in household plumbing and old house paints.

Once penetrated inside human organism by ingestion (drinking or eating), inhalation or skin contact, heavy metals may be responsible for nausea, vomiting, diarrhea or allergic reactions for short term or low-level exposure (Martin and Griswold, 2009). They can also cause severe diseases in the case of long term or chronic high-level exposure, such as reduced growth and development, cancers, organs or nervous system damages and even death (Prabhakar et al., 2012). There are over 50 elements that are classified as heavy metals, including transition metals, some metalloids, lanthanides and actinides. Among them 17 are considered to be both very toxic and relatively accessible. Lead (Pb), mercury (Hg), arsenic (As), and cadmium (Cd) are generally considered as leader elements in human poisoning even at trace level. The general population is mainly exposed to all these metals from air, drinking water and food, fish being a major source of mercury exposure. Moreover, smokers are highly exposed to cadmium (Järup, 2003). Some other heavy metals, including copper (Cu), zinc (Zn), nickel (Ni), cobalt (Co), selenium (Se), and bismuth (Bi) are known to play a vital role in physiological concentrations but can also be toxic in larger doses. Depending on the metal properties, the toxicity target may be different: kidneys or liver are damaged by Cu, Hg, and Pb ions while these latter two also attack central nervous system (Vallee and Ulmer, 1972; Hamilton et al., 1998). Thus, toxicity levels are related to the nature of the metal but also to its biological and biogeochemical roles, both being strongly dependent on its speciation, i.e., the different available physico-chemical forms, namely particulate (size > 1 μm), colloidal (1 nm–1 μm) and dissolved (≤1 nm) species. These latter include free metal ions, simple inorganic complexes and complexes bearing anthropogenic and natural organic ligands (Templeton et al., 2000). Hence speciation information on heavy metals of concern appears to be data of particular relevance (Kot and Namiesńik, 2000).

In 2000, a new European directive (“Water Frame Directive,” WFD, see Table 1) (European Directive 2000/60/EC) particularly pointed out four heavy metals (Hg, Cd, Pb, Ni) and has established their maximal authorized as well as annual average concentration values in surface waters. As a consequence, environmental monitoring of heavy metals is of critical importance for both ecological assessments and public health preservation. In answer there is an urgent need for *in situ*, real-time, and highly-sensitive sensors in order to multiply control points dedicated to early warning pollution alert (Suib, 2013).

**Table 1:** **Environmental Quality Standards for heavy metals (also called WFD)**.

**Substance**	**CAS number**	**EQS-AA^a^ Inside surface waters^b^ nM ^e^ [μg L^−^ 1]**	**EQS-AA^a^ Other surface waters [μg L^−^ 1]**	**EQS MPC^c^ Inside surface waters^b^ nM [μg L^−^ 1]**	**EQS MPC^c^ Other surface waters [μg L^−^ 1]**
Cadmium and its speciation (according to water hardness level ^d^)	7440-43-9	≤ 0.71 (class 1)	1.78	≤ 4 (class 1)	≤ 4 (class 1)
		[0.08]	[0.2]	[0.45]	[0.45]
		0.71 (class 2)		4 (class 2)	4 (class 2)
		[0.08]		[0.45]	[0.45]
		0.8 (class 3)		5.34 (class 3)	5.34 (class 3)
		[0.09]		[0.6]	[0.6]
		1.33 (class 4)		8.09 (class 4)	8.09 (class 4)
		[0.15]		[0.9]	[0.9]
		2.22 (class 5)		13.3 (class 5)	13.3 (class 5)
		[0.25]		[1.5]	[1.5]
Lead and its speciation	7439-92-1	34.7	34.7	Groundless	Groundless
		[7.2]	[7.2]		
Mercury and its speciation	7439-97-6	0.25	0.25	0.35	0.35
		[0.05]	[0.05]	[0.05]	[0.05]
Nickel and its speciation	7440-02-0	341	341	Groundless	Groundless
		[20]	[20]		

a *Environmental Quality Standard—annual average*.

b *Inside surface waters include rivers, lakes and also water masses (artificial or seriously modified) related to them*.

c *Environmental Quality Standard—maximal permissible concentration*.

d *For cadmium and its compounds, EQS—AA values are functions of water hardness according to the five classes as follows: class 1: <40 mg CaCO_3_ L^−1^; class 2: 40–50 mg CaCO_3_ L^−1^; class 3: 50–100 mg CaCO_3_ L^−1^; class 4: 100–200 mg CaCO_3_ L^−1^; class 5: ≥ 200 mg CaCO_3_ L^−1^*.

e *Molar concentrations have been chosen as reference unit for the sake of comparison facility and regarding to the standards of the WFD (Water Frame Directive), even if this latter uses mass concentrations*.

Heavy metals trace detection is mainly performed using spectroscopic techniques: atomic absorption spectroscopy (Kenawy et al., 2000; Pohl, 2009), inductively coupled plasma mass spectroscopy (ICP-MS) (Caroli et al., 1999; Silva et al., 2009), X-ray fluorescence and neutron activation analysis are the most commonly used. Their main advantages are their versatility since they are suitable for a large panel of elements, their sensitivity and their limit of detection (LOD) in the femtomolar range. However they suffer from several major drawbacks: expensive materials are required and qualified operators are needed to perform the multi-step sample preparation and complex analytical procedures, which are unsuitable for on-site and on time measurements necessary to prevent transient phenomena monitoring. Finally, only total metal concentration can be determined, and speciation data can be reached only by associating supplementary extraction and separation techniques such as chromatography to the spectroscopic detection (Feldmann et al., 2009). These additional steps significantly increase the risk of contamination of the sample and some modifications of the speciation may occur during sample storage or handling.

On the contrary electrochemistry represents an interesting alternative due to its numerous advantages. Electrochemical devices are mostly user-friendly since they require simple procedures. They are also reagentless, low cost, and well-suited for miniaturization and automatic *in situ* measurements with minimal sample changes. Thus, contamination by reagents or losses by adsorption on containers are drastically decreased. Electrochemical systems also allow quite fast analyses with experimental data obtained mostly in real time or in a few minutes. Hence, on-line monitoring of water samples becomes possible, providing dynamic data of relevance for biogeochemical survey. Nevertheless specific developments are still required for such applications, particularly to improve sensitivity, LODs and automation. In this way, a large number of electrochemical techniques with different imposed potential or current modulations have been developed such as differential pulse voltammetry (DPV), square wave voltammetry (SWV) or stripping chronopotentiometry (SCP). Electrochemical sensors also allow high temporal resolution measurements to be obtained when associated to flow injection analysis (FIA) or flow electrochemical analysis cells, thus providing continuous *in situ* measurements. Another analytical performance of high relevance with respect to heavy metals detection concerns the selectivity. In complex media, the signal of the analytical target often experiences interferences due to the presence of other species (sometimes other heavy metals). To solve this problem, several surface functionalization strategies have been developed for many years to improve sensors selectivity.

Many pioneering researchers coming from several countries have initiated and intensified works dealing with electrochemical techniques for heavy metals detection and assay in natural media: Jaroslav Heyrovsky (Czechoslovakia) (Heyrovsky, 1960), Joseph Wang (USA) (Wang et al., 1995), Richard G. Compton (UK) (Agra-Gutierrez and Compton, 1998), George Luther III (USA) (Luther III and Ferdelman, 1993), Laura Sigg (Switzerland) (Goncalves et al., 1985), Arben Merkoçi (Spain) (Aragay and Merkoçi, 2012), Marco Mascini (Italy) (Voccia et al., 2012), and so on. French contribution to this topic is also noticeable. This is mainly due to the voluntary policy lead since 1998 and the Aarhus protocol in which France contracted to limit its release of Pb, Cd, and Hg at a lower level than that recorded in 1990 (Commissariat général au développement durable, 2012). This goal was reached before the protocol came into effect in 2003, but the situation is still worrying: over the 2007–2009 period, 25 heavy metals have been detected in more than 10% of the analyses performed in French rivers (Commissariat général au développement durable, 2011), whereas the contamination of mussels and oysters, which constitute a good indicator of coastal water pollution, remained stable over the last 3 decades (Commissariat général au développement durable, 2012). This review provides a survey of French groups' contribution to the development of electrochemical sensors and methods aiming at heavy metals detection. The paper particularly emphasizes the multidisciplinary of French scientific investigations through the description of the electrochemical techniques and the evaluation of the corresponding analytical performances.

### Polarography

#### Classical technique

Polarography has been certainly the most studied and commonly used electrochemical technique throughout the 20th century since the pioneering work of Heyrovsky in 1922 (Heyrovsky, 1922). This is undoubtedly the consequence of the particular properties of the mercury electrode: continuous renewal of the active surface area, wide cathodic potential window due to the high overpotential corresponding to hydrogen evolution, control of the hydrodynamic conditions by means of mercury drop. These characteristics make polarography a very powerful electrochemical technique for the study of inorganic, organic, organometallic, or biological compounds, not only from a theoretical point of view but also for analytical applications. In this frame the assay of heavy metals has been the subject of numerous papers due to the large inclination of mercury to form amalgams with major metal compounds. For concentrations higher than 10^−5^ M linear sweep voltammetry (LSV) on a dropping mercury electrode (DME) or on a static mercury drop electrode (SMDE) generated at the end of a glass capillary is well-suited. For lower concentrations, the faradic current becomes smaller and the double-layer charging current is not negligible anymore. Pulse techniques, i.e., normal pulse (NPV), differential pulse (DPV) and square wave (SWV) voltammetries have been favored to partially suppress the background current and thus improve the LOD. In the case of trace metals detection these potential pulse programs have been associated with anodic (ASV) or cathodic (CSV) stripping voltammetries on a hanging mercury drop electrode (HMDE) inside which the analyte is pre-concentrated by constant potential electrolysis prior to analysis. The resulting methods, i.e., LSASV, DPASV and SWASV and their combination, allow LODs down to 10^−12^ M to be reached (Bard and Faulkner, 2001). For instance Superville et al. assembled an automatic anodic stripping analysis system with a SMDE to undertake a real-time routine analysis of the dynamic behavior of trace metals (Zn, Pb, Cd) in river, pond and seawater (Superville et al., 2011) (see Table 2 for quantitative features). Furthermore a CSV was included to estimate simultaneously the concentration of dissolved oxygen and reduced sulfur species. Magnier et al. perfected a procedure to assay lead and zinc by ASV and copper by CSV in certified reference freshwater and in the French Deûle river, Cu analysis requiring the complexation with 8-hydroxyquinoline (Magnier et al., 2011).

**Table 2:** **Summary of analytical performances and experimental conditions obtained for heavy metals detection in French scientific academic community**.

**Classification**	**References**	**Electrochemical platform**	**Detection medium**	**Technique**	**Analyte(s)**	**LOD**	**Linear concentration range**	**Analyte pretreatment conditions**
**POLAROGRAPHY**								
			Online monitoring river	DPASV	Total Zn	2.91 nM	12.4–23.2 nM	30 s at −1.3V
					Total Pb	0.03 nM	1.7–3.2 nM	60 s at −0.7V
	Magnier et al., 2011	HMDE		DPCSV	Total Cu	0.6 nM	4.9–7.6 nM	30 s at −1.1 V followed by an adsorption step at −0.25 V during 15 s
	Riso et al., 2006	HgFE	Water samples (treated)	SCP	Fe(III)	1.5 nM	NC	6 cycles of: 0.04 V (9 s) and −0.4V (1 s)
	Tanguy et al., 2010	HgFE	Seawater samples (treated)	SCP	Sb(III)	70 pM	depending on sample	300 s at −0.45 V
	Sladkov et al., 2003	HgFE	0.1 M HNO_3_	SWCSV	Se(IV)	0.8 nM	1–1000 nM	300 s at −0.45 V
	Riso et al., 1997	HgFE	Seawater	SCP	Cu	0.7 nM	NC	15 min at −1.1 V
					Pb	14 pM		
					Cd	9 pM		
	Cugnet et al., 2009	SPμEAs	0.2 M acetate (pH = 4.5)	SWASV	Cd(II)	11.6 nM	11.6–89 nM	300 s at −1 V
	Parat et al., 2011a	HMDE	0.1 M KNO_3_	AGNES-SCP	Zn^2+^	4 nM	at least 25–100 nM	1400 s total with complex procedure
					Cd^2+^	2.9 nM		
					Pb^2+^	4.1 nM		
	Parat et al., 2007	Membrane and Hg film SPE	0.2 M acetate (pH = 4–7)	SWASV	Cd(II)	2 nM	5–100 nM	60 s at −1 V
	Munteanu et al., 2009	Mercury monolayer carbon fiber electrode	NC	SWASV	Pb(II)	80 fM	1–10 pM	1 s at −1.2 V
	Parat et al., 2011b	Hg film SPE	0.2 M acetate (pH = 4.6)	SSCP	Cd	2.2 nM	NC	60 s at −1 V
	Zaouak et al., 2010a	Hg film SPE	0.2 M acetate (pH = 4.5)	SWASV	Cd	1.78 nM	1.78–356 nM	60 s at −1 V
**Bi FILMS**								
	Guo et al., 2005	GC/BiFE	Milk vetch in 0.2 M KSCN	ASV	Zn(II)	9.6 nM	500–3000 nM	120 s at −1.4 V
	Legeai et al., 2005	GC/BiFE	0.125 M HNO_3_ + 0.04 M H_2_NSO_3_H	DPASV	Cd(II)	~10 nM	20–1000 nM	1200 s at −0.95 V
	Legeai et al., 2006	Cu/Bi film electrode	0.01 M ammonia buffer (pH = 9)	SWASV	Ni^2+^	NC	10–1000 nM	900 s at −0.7 V
	Legeai and Vittori, 2006	Cu/Nafion/Bi electrode	0.01 M NaCl + 0.001 M NaHCO_3_	DPASV	Cd^2+^	6.05 nM	17.8–107 nM	300 s at −0.95V
					Pb^2+^	3 nM	9.65–86.9 nM	
	Urbanova et al., 2010	Highly porous Bi film electrodes	0.1 M acetate buffer (pH = 4.5)	DPASV	Cd(II)	5.34 nM	178–1160 nM	90 s at −0.95 V
					Pb(II)	6.27 nM	96.5–627 nM	
	Zaouak et al., 2009	Bi-Coated SPμE	0.2 M acetate buffer (pH = 4.5)	SWASV	Cd(II)	11.6 nM	45–400 nM	120 s at −1 V
	Lu et al., 2010	Bi doped carbon SPE	Air	SWASV	Pb(II) as vapor	1 ng	10–80 ng	120 s at −1.2 V
**CARBON ELECTRODE MATERIALS**								
**DLC/GC**								
	Feier et al., 2012	Graphite felt	0.1 M NaBF_4_	LSASV	Zn(II)	50 nM	1–100 μM	300 s at −1.4 V
	Nasraoui et al., 2009	Graphite felt	0.1 M LiClO_4_	LSASV	Pb(II)	1 nM	10–500 nM	300 s at −1 V
	Khadro et al., 2009	GC electrode	0.1 M HCl	DPASV	Ni(II)	2.56 nM	8.52–9370 nM	60 s at −1 V
			0.1 M acetic buffer		Hg(II)	0.15 nM	0.5–1740 nM	
	Khadro et al., 2011	B-doped DLC	Acetate (pH = 4.2)	SWASV	Cd(II)	4.83 nM	4.83–121 nM	90 s at −1.3 V
					Pb(II)	8.9 nM	8.9–222 nM	
					Ni(II)	34.1 nM	34.1–256 nM	
					Hg(II)	4.99 nM	5–125 nM	
**BDD**								
	Le et al., 2012	BDD	acetate pH = 5.2	SWASV	Pb(II)	19.3 nM	96.5–480 nM	600 s at −1 V
	El Tall et al., 2007	BDD	0.01 M acetate	DPASV	Cu(II)	14.2 nM	47–315 nM	60 s at −1.9 V
					Pb(II)	5.55 nM	18–217 nM	
					Zn(II)	25.5 nM	77–305 nM	
					Cd(II)	3.2 nM	11–222 nM	
	Sbartai et al., 2012	BDD	Potassium citrate / HCl	DPASV	Cd(II)	3.29 nM	NF	20 s at −1.7 V
					Pb(II)	26.5 nM		
					Ni(II)	116 nM		
					Hg(II)	11.5 nM		
**ISEs**								
**CALIXARENE**								
	Yaftian et al., 2006	Calix[4]arene	Complex (pH = 3.5–5)	SCP	Pb(II)	1.4 μM	10μM–10 mM	No accumulation and electrolysis
	Yaftian et al., 2007	Calix[4]arene	Complex (pH = 3–7)	SCP	Pb(II)	4 nM	10 nM–100 μM	No accumulation and electrolysis
**CHALCOGENIDE**								
	Cali et al., 2002	Cu-As-S	KNO_3_	SCP	Cu(II)	1 μM	2 μM–10 mM	No accumulation and electrolysis
	Essi and Pradel, 2011a,b	Cu-Ag-S	Complex (pH = 3–5)	SCP	Cu(II)	1 μM	NC	No accumulation and electrolysis
	Mear et al., 2005	Ge_28_Se_60_Sb_12_	KNO_3_ (pH = 3)	SCP	Cd(II)	1 μM	1 μM–10 mM	No accumulation and electrolysis
**CMEs**								
**MINERALS**								
	Walcarius et al., 1999a	Silica modified CPE	0.2 M HNO_3_	SWASV	Cu(II)	2 nM	5 nM–5 μM	600 s accumulation followed by 30 s at −0.5 V
	Walcarius et al., 2000	Silica-modified electrode	0.1 M HNO_3_	SWASV	Hg(II)	50 nM	200 nM–10 μM	600 s accumulation at open circuit followed by 60 s at −0.5 V
	Walcarius et al., 1999b	Several silica/hybrid CPE with amine functionalization	0.05 M acetic acid + 0.05 M NaNO_3_	LSASV	Cu(II)	NC	unclear	Several accumulation time and 240 s at −0.4 V
	Etienne et al., 2001	Organically modified silica	0.1 M sodium acetate	SWASV	Cu(II)	3 nM	50–200 nM	60 s at −0.5 V
	Sayen et al., 2003	Carnosine silica hybrid material modified CPE	0.1 M NaNO_3_ + 0.01 M HNO_3_	DPASV	Cu(II)	4 nM	50–1000 nM	90 s at −0.5 V
	Walcarius and Sibottier, 2005	Amine-functionalized porous silica films on Au	0.1 M HNO_3_ + 0.1 M NaNO_3_ in 95% ethanol	DPASV	Cu(II)	40 nM	0.1–10 μM	600 s accumulation followed by 60 s at −0.4 V
	Etienne et al., 2007	Surfactant-templated thiol-functionalized silica thin films	0.5 M HCl	DPASV	Ag(I)	6 nM	0.2–10 μM	960 s accumulation followed by 60 s at −0.6 V
	Sanchez and Walcarius, 2010	GC/MTTZ	0.1 M HCl	SWASV	Hg(II)	2 nM	50 nM–1 μM	300 s at −0.4 V
	Walcarius et al., 1998	Mesoporous pure silica modified carbon paste electrode	0.2 M HNO_3_	SWASV (or CV for larger amounts)	Cu(II)	30 nM	NC	300 s accumulation followed by 60 s at −0.5 V
					Hg(II)	50 nM	NC	
	Tonle et al., 2005	Clays grafted with organic chelating groups (thiol or amine) modified CPE	0.1 M HNO_3_	DPASV	Hg(II)	68 nM (thiol) 87 nM (amine)	100–700 nM	180 s accumulation followed by 60 s at −0.4 V (or −0.6 V depending on the medium)
	Tonle et al., 2011	Thiol-functionalized clay modified CPE	0.2 M HNO_3_	SWASV	Pb(II)	60 nM	0.3–10 μM	600 s accumulation followed by 60 s at −0.9 V
	Tchinda et al., 2007	GC/PCH-SH	0.1 M HCl + 5% thiourea	DPASV	Hg(II)	0.4 nM	4–20 nM and 50–80 nM	1200 s accumulation followed by 180 s at −0.7 V
**MACROCYCLIC COMPOUNDS**								
	Rouis et al., 2013	β-ketoimine calix[4]arene on ITO	0.05 M ammonium acetate (pH = 7)	Impedance	Hg^2+^	NC	0.1 nM–0.5 μM	
	Goubert-Renaudin et al., 2009a	Cyclam-functionalized silica CPE	3 M HNO_3_	SWASV	Cu(II)	0.8 nM	2–100 μM	1800 s accumulation followed by 60 s at −0.5V
	Goubert-Renaudin et al., 2009b	(TETAM) grafted to silica gel and ordered mesoporous silica	0.1 M ammonium acetate buffer (pH = 7)	SWASV	Pb(II)	2.7 nM	10–100 nM	900 s accumulation followed by 60 s at −0.8 V
	Nasraoui et al., 2010a	TETRAM-modified graphite felt electrode	0.1 M aqueous solution of LiClO_4_	LSASV	Pb(II)	25 nM	100–250 nM	around 1800 s accumulation followed by 300 s at −1 V
	Nasraoui et al., 2010b	Cyclam-modified graphite felt	0.5 M H_2_SO_4_	LSASV	Pb(II)	25 nM		several accumulation time followed by 300 s at −1 V
	Parat et al., 2006	Hg film modified SPE	0.1 M KNO_3_	LSASV	Cd(II)	6 nM	NC	120 s at −1 V
					Pb(II)	8 nM		
	Betelu et al., 2007	Hg film + membrane modified SPE	0.01 M NaHCO_3_	LSASV	Cd(II)	NC	NC	120 s at −1 V
					Pb(II)			
**POLYMERS**								
	Heitzmann et al., 2007	Poly(pyrrole-EDTA like) film	0.1 M buffer (pH = 5)	SWASV	Cd(II), Pb(II) and Cu(II)	NC	NC	600 s accumulation followed by 40 s at −1.2 V for Pb(II) and −0.9 V for Cu(II)
					Hg(II)	0.5 nM	NC	
	Buica et al., 2009a	Poly(EDTA-like) Film	0.1 M acetate buffer (pH = 4.5)	DPASV	Cu(II)			600 s accumulation followed by 60 s at −0.4 V
	Buica et al., 2009b	Poly(pyrrole-EDTA) modified electrode	0.1 M acetate buffer (pH = 4.5)	DPASV	Hg(II)	10 nM (imprinted polymers)	10–1000 nM (imprinted polymers)	600 s accumulation followed by 180 s at −1.8 V
					Pb(II)	0.5 nM	10–1000 nM	600 s accumulation followed by 40 s at −0.9 V
					Cu(II)	5 nM	25–250 nM	
	Heitzmann et al., 2005	Poly(pyrrole-malonic acid) film modified carbon electrode	0.2 M acetate buffer (pH = 4.4)	SWASV	Hg(II)	50 nM	NC	
					Cd(II)	0.2 μM	1–10 μM	600 s accumulation followed by 40 s at −1.1 V
	Pereira et al., 2011	Complexing polymer films	0.1 M acetate buffer (pH = 4.4)	SWASV	Pb(II)	0.5 nM	10–1000 nM	600 s accumulation followed by 40 s at −0.9 V or −1.1 V for Cd(II)
					Cu(II)	5 nM	25–250 nM	
					Hg(II)	100 nM	100–1000 nM	
					Cd(II)	500 nM	100–10000 nM	
	Rivas et al., 2006	Complexing polymer films	0.1 M acetate buffer (pH = 4.8)	SWASV	Pb(II)	NC	0.01–5 mM	600 s accumulation followed by 40 s at −0.6 V
	Bessbousse et al., 2011	Nanoporous β-PVDF membrane electrode	0.1 M sodium acetate	SWASV	Pb(II)	0.63 nM	NC	30 min equilibrium followed by 100 s at −0.8 V
	Zejli et al., 2007	Polythiophene film	0.2 M KNO_3_ (pH = 5)	DPASV	Ag(I)	0.56 μM	0.65–9.3 μM	120 s at −0.5 V
	Yasri et al., 2011	GC/PEDOT:PSS	HCl (pH = 2.2)	CA	Pb(II)	0.19 nM	2–100 nM	30 s at −0.65 V
**NPs**								
	Ottakam Thotiyl et al., 2012	Au/MPS-(PDDA-AuNPs)	Phosphate buffer (pH = 8)	DPASV	As(III)	0.48 μM	NC	
	Hezard et al., 2012a	GC + AuNPs	0.01 M HCl	SWASV	Hg(II)	0.42 nM	0.64–4 nM	300 s at 0 V
	Hezard et al., 2012b	GC + AuNPs	0.01M HCl	SWASV	Hg(II)	0.4 nM	0.8–9.9 nM	300 s at 0 V
**BIOSENSORS**								
	Chouteau et al., 2004	Alkaline phosphatase	10 mM Tris-HCl buffer (pH = 8.5) / 1 mM MgCl_2_	Conductometry	Cd^2+^	8.9 nM	NC	
	Chouteau et al., 2005	Alkaline phosphatase Acetylcholinesterase	10 mM Tris-HCl buffer (pH = 8.5) / 1 mM MgCl_2_	Conductometry	Cd^2+^	89 nM	NC	30 min incubation
					Zn^2+^	0.15 μM		
	Tekaya et al., 2013	Alkaline phosphatase	5 mM HEPES buffer (pH = 8.1)	Conductometry	Cd^2+^	10^−20^ M	NC	24 h incubation
					Hg^2+^			
	Soldatkin et al., 2012	Invertase, mutarotase, glucose oxidase	5 mM phosphate buffer (pH = 6.5)	Conductometry	Hg^2+^	25 nM	NC	20 min incubation
					Ag^+^	100 nM		
	Mohammadi et al., 2005	Invertase, mutarotase, glucose oxidase	0.1 M phosphate buffer (pH = 6)	Amperometry	Hg(II)	NC	10 nM–1 μM	20 min incubation at pH = 4
	Gayet et al., 1993	L-lactate dehydrogenase L-lactate oxidase	0.1 M Tris buffer (pH = 9)	Amperometry	Hg(II)	1 μM	NC	5 min incubation
					Ag^+^	0.1 μM		
					Cd^2+^	10 μM		
					Zn^2+^	10 μM		
					Pb^2+^	50 μM		
					Cu^2+^	250 μM		

^*^All parameters given are the ones for the best LODs.

The need for determination of very low concentrations has favored the development of specific electrochemical techniques with new potential perturbation modes providing high resolution and/or improved sensitivity. In this way Zlatev et al. particularly emphasized the advantages of differential alternative pulses voltammetry (DAPV) on HMDE to analyse mixtures of species exhibiting very close half-wave potentials (like Pb^2+^ and Tl^+^ or Co^2+^ and Ni^2+^) or species couples with high concentration ratios (Zlatev et al., 2006) for which the analysis by DPV is hampered by complete peaks overlapping. DAPV takes advantage of the high resolution power of the second-order voltammetric techniques (as radio-frequency polarography) combined with the high sensitivity and instrumental simplicity of DPV or SWV. DAPV principle is based on the superimposition on the main electrode potential E of a pair of single successive rectangular pulses characterized by small, equal amplitudes (<RT/nF) and durations (from 1 to 100 ms) but opposite polarities. The overall current recorded at this potential E corresponds to the deviation of the average of the corresponding cathodic and anodic amperometric responses. Thus the resulting current-potential curve exhibits the typical shape of a first-order peak derivative passing three times through zero (for potential ranges corresponding to residual and diffusion-limited currents and for the electrode potential equal to the half-wave potential) with peak amplitudes proportional to the electroactive species concentration. The resolution power of DAPV was highlighted through the analysis of a solution containing Pb^2+^, Tl^+^, In^3+^, and Cd^2+^. The sensitivity and the LOD (54 nM) were found to be similar to those obtained using classical DPV but with species having half-wave potentials difference in the range from 28 to 50 mV and concentration ratios from 1:1 up to 80:1 without any preliminary preparation of the sample.

#### Mercury film

Despite very good analytical performances in terms of sensitivity and stability of the response vs. time, the low vapor pressure and the high toxicity of mercury encouraged extensive researches on polarographic methods involving reduced amounts of mercury. One way consists in thin film mercury electrodes (TFME) electrodeposited on solid state materials like glassy carbon (GC). The group of Riso used SCP with low constant current for the quantification of Fe(III) in estuarine and coastal filtered waters (Riso et al., 2006). The procedure was proved to be highly sensitive but analysis required several pre-treatment steps, i.e., filtration of the sample and complexation with solochrom violet. They also succeeded in detecting ultra-trace (70 pM) Sb(III) in seawater by using the same electrochemical method. The application of a double electrolysis potential during the pre-concentration step allowed the analysis to be independent from the Cu level (Tanguy et al., 2010).

For the determination of Se(IV), insufficient reproducibility and sensitivity of Hg film was observed by Sladkov et al. (2003). This problem was overcome by incorporating Cu(II) ions during the plating procedure on GC electrode surface. The metallic Cu dissolved in the Hg film was found to play an important role in peak current enhancement. A LOD 0.8 nM was reached by SWCSV and the relative standard deviation was 5.2% (*n* = 5) for 1 μM Se(IV).

A potentiometric stripping method has been proposed by Riso et al. for the simultaneous measurement of Cu, Pb, and Cd in ocean waters (Riso et al., 1997). The mercury coating was electrodeposited *in situ* on a GC rotating electrode at the beginning of each analysis by applying a potential step at −1.1 V/SCE for 10 min. Then an electrolysis-stripping cycle was carried out. Metals concentrations were compared with a reference standard solution containing all three metals. The obtained LODs were 0.7 nM, 15 pM and 9 pM for Cu, Pb, and Cd, respectively. However, it has to be noticed that the total duration of the analysis was quite long, about 75 min.

In order to approach solid mercury-free electrodes, Munteanu et al. worked on the electrodeposition of a mercury monolayer by constant potential electrolysis with increasing electrolysis time (Munteanu et al., 2009). An exceptional sensitivity for Pb^2+^ assay was obtained when the mercury monolayer-on-carbon electrode was used with fast (*v* > 1 kV/s) ASV. This result was revealed to be due to the ionization of Pb atoms in the mercury layer, which catalyzes the oxidation of atomic hydrogen adsorbed on the Hg layer. A remarkable LOD of 80 fM was recorded on a cylinder electrode with a 1 s preconcentration time.

#### Mercury screen printed electrode (HgSPE)

Potin-Gautier's group has developed an alternative strategy based on mercury micro arrays screen printed electrodes (SPE). This micro system allowed mass transport of the analytes to be enhanced compared to macro systems. This device was successfully tested for Cd^2+^ detection in synthetic and river samples, providing a LOD of 11.6 nM using SWASV (Cugnet et al., 2009). More recently SCP was implemented as the second stage of the electrochemical technique “Absence of Gradients and Nernstian Equilibrium Stripping” (AGNES-SCP) for the determination of free metal concentration, namely Zn^2+^, Cd^2+^, and Pb^2+^, with both an HMDE and a SPE (Parat et al., 2011a). The results showed higher sensitivities and lower LOD and LOQ (in the nanomolar range) with SPE, which was linked to a higher product of mercury volume times the gain (AGNES parameter). Finally, SPE was modified by a microwell for the assay of labile Cd^2+^, thus reducing the sample volume down to 200 μL. A LOD of 2 nM was reached using SWASV with only a 60 s preconcentration step. Unfortunately, the performances of this electrode were pH-dependent out of the pH range 4–7 (Parat et al., 2007). Nevertheless all these mercury-based techniques are promised to disappear in a few years since no mercury will be authorized from 2015 (European Directive 2008/105/EC).

### Bismuth

Bismuth film electrode (BiFE) is often considered to be a good alternative to Hg electrode and has been extensively used for electroanalysis. More “environmentally friendly” and less toxical than Hg, Bi is considered to be a safe material, as it is a non-carcinogenic element (except for foetus and embryo) (Svancara et al., 2010). However, in high doses, it presents similar toxicity to other heavy metals apart from these effects are much more reversible. The main advantage of Bi with respect to trace analysis is its capability to form binary or multi-component alloys with numerous other heavy metals (Svancara et al., 2010). It has also the particularity to be the most diamagnetic metal, thus avoiding conductance problems. Another interest of Bi compared to Hg is its insensitivity to dissolved oxygen, thus making unnecessary any deaeration step. Most of the time, the Bi film is plated before analysis onto the electrode by potentiostatic reduction of a Bi(III) solution (Wang et al., 2001), although codeposition during trace metal reduction has been also reported (Wang et al., 2000). BiFE performances compare favorably with Hg electrodes, affording high sensitivity and well-defined stripping signals. For instance, Guo et al. compared the anodic stripping voltammetric response of HgFE and BiFE obtained in a 10 mM Zn(II) solution (Guo et al., 2005). The BiFE presented a well-defined and higher stripping peak compared to HgFE. Its sensitivity was found to be twice higher than that obtained on HgFE. This electrode was then used to detect zinc contained in milk vetch used in traditional Chinese medicine. The response was linear in the range from 0.5 to 3 μM and a LOD of 10 nM was reported. Nevertheless, it has to be noticed that the cathodic limit of the potential window is higher than on Hg.

In 2005, Legeai et al. proposed an interesting alternative to the classical Pt or GC substrates by electrodepositing Bi film onto Cu since the adherence of the film was found to be better in this latter case (Legeai et al., 2005). By using DPASV as the detection method, this BiFE exhibited very good performances toward Cd^2+^ assay in acidified tap water with a linear concentration range between 10^−8^ and 10^−6^ M. It was also successfully tested for the simultaneous determination of Cd^2+^, Zn^2+^ and Pb^2+^ ions at 10^−5^ M. Good accuracy (<5%) was confirmed by comparison of the electrochemical data with those obtained by ICP-MS and mercury-drop electrode on aquatic plant extracts. These results were later generalized to Ni^2+^ analysis, by using adsorptive stripping voltammetry and dimethylglyoxime as the complexing agent (Legeai et al., 2006). However, BiFEs suffer a major drawback since the formation of Bi hydroxides, which occurs at pH higher than 4.3, makes the analytical results irreproducible due to film surface changes. To overcome this problem, Legeai et al. used a Nafion-coated Cu substrate for Bi electrodeposition and observed that the Bi particles which were incorporated into the membrane did not experience any hydroxylation reaction (Legeai and Vittori, 2006). This system was successfully used for Cd^2+^ and Pb^2+^ detection and exhibited good stability (only 10% decrease after 23 days).

**Figure 1:**
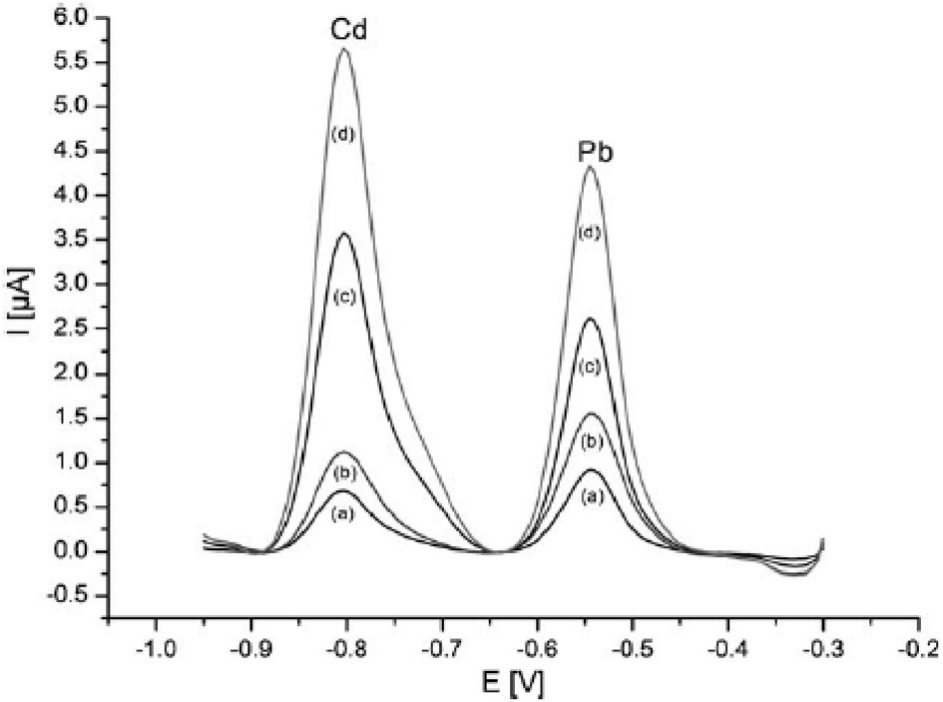
**DPASV voltammograms for increasing level of Cd and Pb in the 20–130 ppb range on porous Bi film (gold substrate) electrode**. Reprinted with permission from Urbanova et al. (2010). Copyright 2010 Wiley-VCH.

In a very close approach, Urbanova et al. succeeded in increasing the active surface area of the electrode by depositing Bi onto a polystyrene spheres template (Urbanova et al., 2010). After polystyrene dissolution by toluene washing, the resulting ordered porous film exhibited an original three-dimensional structure which significantly enhanced the analytical performances. These latter were found to be strongly correlated to the Bi film thickness. By using the thickest porous film (2.2 μm thickness) and DPASV method, linear concentration ranges between 178 nM and 1.16 μM for Cd^2+^ ions and between 96.5 and 627 nM for Pb^2+^ ions were obtained (Figure 1). The LODs were 5.3 nM and 6.3 nM respectively, with a pre-concentration time of 300 s. The standard deviations were lower than 4% in both cases.

Zaouak et al. reported comparable performances by using a Bi film electrodeposited on carbon screen-printed microband electrode and SWASV (linear range from 10 to 400 nM and LOD 5.34 nM with 8% reproducibility and 3% repeatability in synthetic solution) (Zaouak et al., 2009). This system was successfully tested on non-treated river water sample but exhibited poor stability in this case (4% decrease in 10 min) due to both Bi film hydroxylation and biofouling. To overcome this drawback, the authors suggested the renewing of the microband after each measurement.

Finally, the best performances of Bi electrodes were reported by Lu et al. for trace metal vapors detection (Lu et al., 2010). In this case, Bi powder was simply mixed with carbon ink and the resulting Bi SPE was covered with a 0.1 mm thickness hydrogel layer by immersion in an agarose solution. This latter acted both as solid electrolyte and preconcentration agent, allowing Pb vapor concentrations between 48 and 386 pM to be determined with a LOD around 4.8 pM. Similar results were obtained with Zn and Cd.

### Carbon electrode materials

Nowadays, a particular attention is paid to more ecofriendly solid electrodes. GC or graphite felts are the easiest carbon based material to obtain (structured with sp^2^ carbon atoms). Alternatively, Boron doped diamond (BDD) or diamond like carbon (DLC) with sp^3^ carbon atoms structure are very interesting for their specific properties (McCreery, 2008).

#### Graphite

Graphite felts are porous electrodes made of 10 μm diameter carbon fibers. They generally present a high specific surface area (around 1200 cm^2^ g^−1^) and a high void volume (around 90%) which enhances hydrodynamics, thus improving the LOD of the resulting sensor. Feier et al. exploited these particular properties to elaborate a flow sensor for Zn^2+^ trace analysis (Feier et al., 2012). A LOD of 500 nM was reached by LSASV with a 5 min duration cathodic preconcentration step. Nevertheless the selectivity toward Zn^2+^ detection was strongly affected by several interfering species such as Fe^3+^, Cu^2+^, Co^2+^, and Ni^2+^. Nasraoui et al. elaborated a Pb sensor by using a similar flow cell and the same electrochemical method (Nasraoui et al., 2009). A LOD as low as 1 nM was obtained for an overall analysis time of 11 min.

In order to increase the sensitivity, Khadro et al., elaborated a SPE involving active surface carbon deposit (1 mm diameter) for the assay of Hg^2+^ and Ni^2+^ (Khadro et al., 2009). Using DPASV as mutual detecting mode, the sensitivity was respectively 10 and 20 times higher than those obtained with a traditional batch electrochemical cell including a three times higher diameter working electrode. Consequently the LODs decreased to 0.15 and 2.56 nM for Hg^2+^ and Ni^2+^, respectively. Despite satisfactory performances, the main drawback concerned electrode fouling which obliged the regular regeneration of the surface between analyses and reduced the electrode life-time: Nasraoui et al. observed a decrease about 10 and 20% of the electrochemical signal after the second and third regeneration, respectively (Nasraoui et al., 2009).

#### Amorphous nitrogenous carbon thin film (a-CNx)

**Figure 2:**
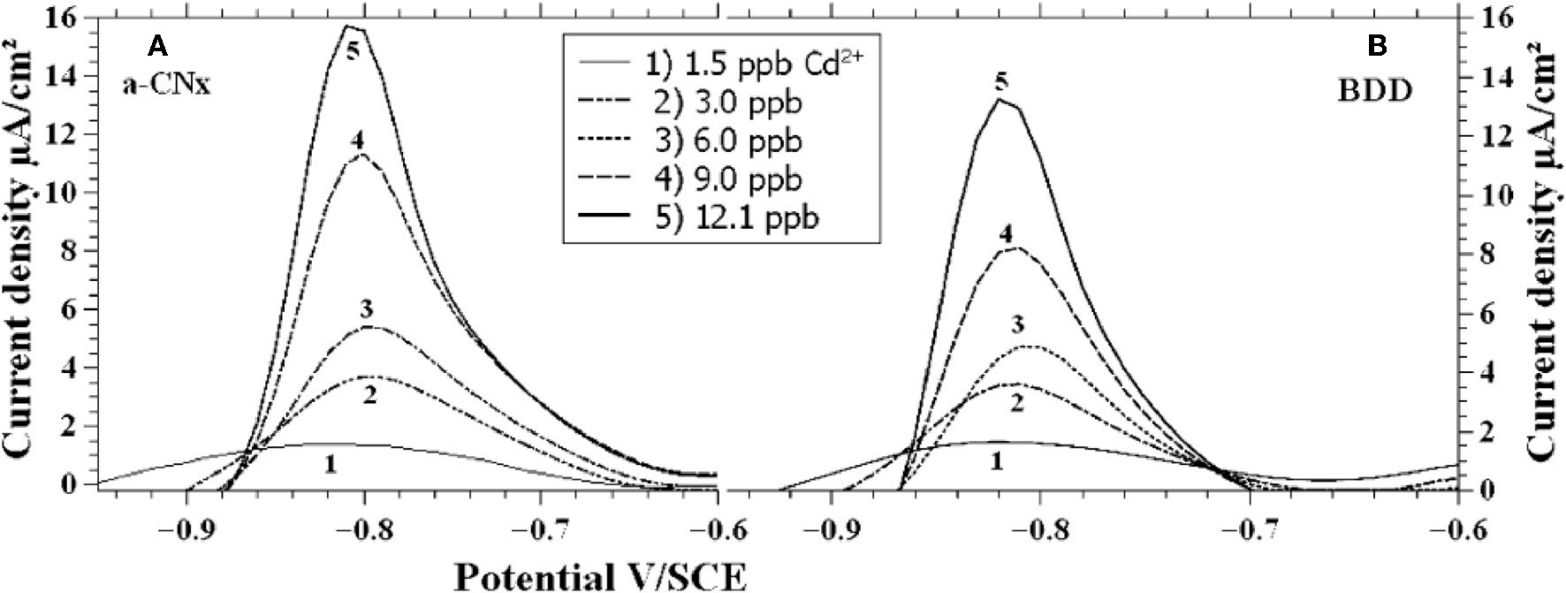
**Voltammograms for a-CNx (A) and BDD (B) in solutions containing different Cd^2+^ concentrations**. Reprinted with permission from Seck et al. (2012). Copyright 2012 Wiley-VCH.

a-CNx thin film is a carbon-based material (around 80% sp^2^ carbon) including nitrogen atoms. It is synthesized by a radio-frequency magnetron sputtering technique. The amount of nitrogen incorporated into the film can be controlled by the composition of the plasma (N_2_/Ar ratio) used for the deposition. The main property of a-CNx electrodes concerns their broad electrochemical window, which makes it particularly suitable for electrodetection of many species. This was exploited by Seck et al. for the simultaneous assay of Cd^2+^ and Cu^2+^ (Seck et al., 2012) (Figure 2). The presence of Cu modified the peak current related to Cd^2+^ as compared to Cd^2+^ detection in Cu-free solution. Anyway the a-CNx electrode allowed the detection of 2 ppb of Cd^2+^ with concentration of Cu^2+^ up to 140 ppb.

#### Diamond like carbon

Diamond like carbon (DLC) is a carbon-based material containing a mixture of sp^2^ (graphite) and sp^3^ (diamond) carbon phases. Several methods have been developed to produce DLC films: plasma enhanced chemical vapor deposition (CVD) techniques, ion beam, filtered cathodic vacuum arcs. DLC exhibits some unique properties, such as high elastic modulus, high mechanical hardness, very low surface roughness and chemical inertness. Khadro et al. used DLC doped with boron to improve its conductivity (Khadro et al., 2011). The resulting electrode was exploited for the simultaneous assay of many heavy metals in water, namely Cd^2+^, Pb^2+^, Ni^2+^, and Hg^2+^, in concentration ranges up to 200 nM. LODs of 8.9, 4.8, 34, and 5 nM were reached, respectively.

#### Boron doped diamond

**Figure 3:**
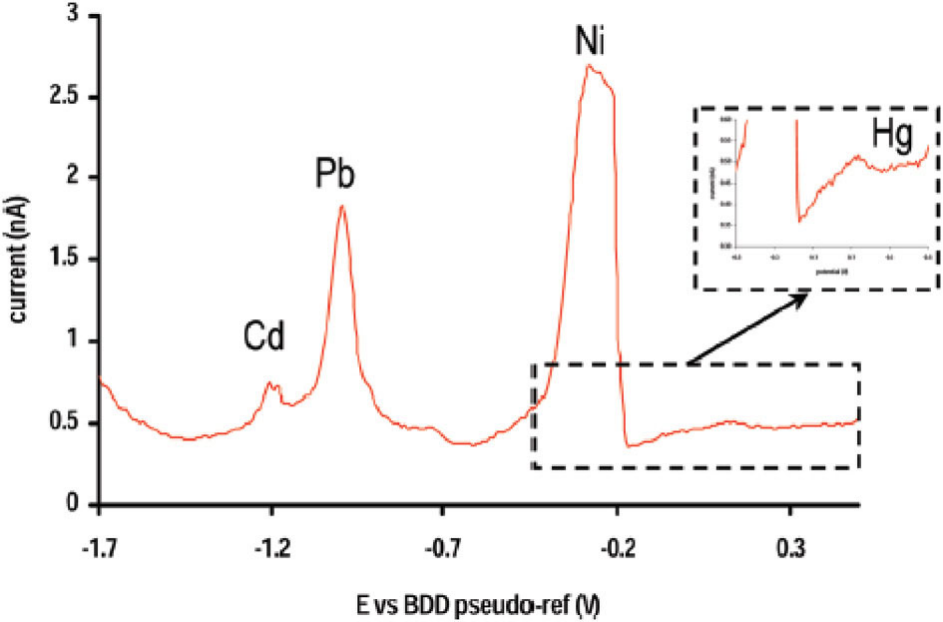
**DPASV obtained with a BDD micromachined microcell on a standard solution of Cd (20 nM), Ni (38 nM), Pb (11 nM) and Hg (0.55 nM)**. Reprinted with permission from Sbartai et al. (2012). Copyright 2012 American Chemical Society.

Boron doped diamond (BDD) is the most recent carbon-based material used for electroanalytical purpose. Diamond films can be deposited using CVD systems involving activation of gases by either microwave plasma or a hot filament. Traditionally electrical conductivity of diamond films is obtained through doping with boron (p-type behavior). The advantages of such material are manifold compared to previous carbon-based ones: BDD has an extremely high chemical stability, presents a wide potential window in aqueous media (−1.35 to +2.3 V/NHE at 0.1 mA cm^−2^ in 0.5 M H_2_SO_4_) and generates a low background current. Moreover it is extremely resistant to fouling phenomena, thus making its surface state very reproducible with time. Unlike graphite felt, BDD exhibits however lower specific area. In order to increase the sensitivity two major ways have been envisaged. In the one hand, Le et al. associated a BDD electrode with a microelectrodialyser as a preconcentration step (Le et al., 2012). The corresponding device allowed the assay of Pb^2+^ ions with a linear concentration range between 96 and 490 nM and a LOD of 19 nM. The same analytical device was used for the simultaneous detection of Zn, Cd, Pb, and Cu (El Tall et al., 2007). Quantification was possible for the first three heavy metals but the presence of Cu caused interferences. Compared to GC, BDD electrode exhibited an enhanced sensitivity (3 or 5 times) and a longer lifetime in real samples (El Tall et al., 2007). In the other hand, Sbartai et al. developed a new electrochemical microcell micromachined by a femtosecond laser for the simultaneous detection of Cd, Ni, Pb, and Hg (Sbartai et al., 2012) (Figure 3). Reduction of the electrode size resulted in mass transport amplification. LODs of 0.4, 6.8, 5.5, and 2.3 nM were thus obtained, respectively. Quantitative results were recorded for concentrations up to 200 nM. These performances are comparable to those obtained on DLC by Khadro et al. for Pb^2+^ and more accurate for Hg^2+^, Cd^2+^, and Ni^2+^ (Khadro et al., 2011). However, a non-linear calibration curve for Hg was obtained in the former paper, which can be explained by the presence of Cl^−^ ions in the electrolyte solution, leading to Hg_2_Cl_2_ formation at the electrode surface.

New carbon materials like a-CNx, DLC or BDD which exhibit high chemical stability and offer a wide potential window hold significant promises for electronalytical applications. Moreover, there is a wealth of opportunities for nanoscale electrochemical devices based on carbon materials. Nevertheless, interceptions between cations go through either the development of specific calibrations or the chemical modification of the electrode.

### Ion selective electrodes

Ion-selective electrodes (ISEs) are potentiometric sensors that involve a selective membrane which minimizes matrix interferences (Bobacka et al., 2008). The response of these sensors is based on an equilibrium state complexation reaction between the analyte and the probe with kinetic properties strongly depending on the membrane composition. The potentiometric measurement as well as the nature of the molecular interactions generally allow LODs around 1 μM to be reached and a linear concentration range from 10^−5^ to 10^−2^ M in a pH window from 3 to 6. Innovative changes have been made in recent past years to improve these analytical performances. In this context several functionalized materials have been used, including glass, liquid or polymer membranes. In the latter case an ionophore is generally used as the sensing platform. In this way, Yaftian et al. first synthesized phosphorylated calix[4]arene coated on a graphite electrode for the assay of Pb traces (Yaftian et al., 2006). This sensor exhibited a particularly quite long lifetime (up to 8 weeks) and a relatively short response time (17 s). This latter was significantly shortened to 7 s by using hexahomotrioxacalix[3]arene as the ionophore, due to fast exchange kinetics complexation-decomplexation processes (Yaftian et al., 2007). Furthermore the concentration range covered four decades (between 10^−8^ and 10^−4^ M). The specificity of this ISE toward Pb^2+^ was successfully tested in synthetic solution in the presence of 22 interfering species. Nevertheless no measurement was done in real sample water. In both cases, the electrode was also used in potentiometry for the titration of Pb^2+^ solution using a standard EDTA solution.

Glassy materials represent a good alternative, particularly in micro-sensor fabrication. Beyond the advantages of all solid state devices, vitreous materials are well-suited for the production of homogeneous thin layers which allow potentiometric measurements to be done despite their poor conductivity. Thereby, several types of solid state membranes have been synthesized like the widely used chalcogenide one, in which the conduction over the membrane is provided by halides or metallic ions. Chalcogenide glasses exhibit better chemical durability in acidic media, and in many cases, afford better selectivity and reproducibility than arene ionophores. Several French research teams have investigated such ISEs toward Cu(II) determination. Cali et al. used chalcogenide glassy-crystalline Cu-As-S alloys (Cali et al., 2002). The resulting sensor exhibited a very short response time (1–3 s) and a LOD close to 10^−6^ M with a linear concentration range between 10^−6^ and 10^−2^ M. These results are available within a pH range from 2 to 6. Similar analytical performances were obtained by Essi et al., with a Cu-Ag-S thin film for the assay of Cu(II) and Ag(I) ions (Essi and Pradel, 2011a). They were satisfactorily compared with those obtained by ICP-MS in real samples. Furthermore the specificity of the sensor was not damaged by the simultaneous presence of Ca^2+^, Mg^2+^, Pb^2+^, Cd^2+^, and Zn^2+^ (Essi and Pradel, 2011b). Mear et al. investigated a thin film of Ge_28_Se_60_Sb_12_ chalcogenide glass including Cu in order to quantify Cu(II) ions (Mear et al., 2005). A linear range was found between 10^−5^ and 10^−3^ M with a LOD of 3 μM. The concentration range was enlarged between 10^−6^ and 10^−2^ M for Cd(II) ions assay by coupling the membrane electrode with a pre-concentration module.

### Chemically-modified electrodes

From the analytical point of view, the wide and increasing success of chemically modified electrodes (CME) may be explained by the offered possibility to purposely design the surface of conventional electrodes. By combining the intrinsic properties of the modifier and a given electrochemical reaction, CMEs exhibit significantly improved response compared to unmodified electrodes (Murray et al., 1987; Gilmartin and Hart, 1995; Cox et al., 1996). For heavy metals trace detection, the modification plays a critical role especially during the preconcentration step, by favoring selective and enhanced accumulation, thus leading to higher sensitivity and lower LODs (Arrigan, 1994). During the detection step, the modification also often favors the electron transfer kinetics. The modifier may be a mineral such as silica or clay, a polymer, an inorganic or organic compound or a metal nanoparticle based material. Depending on its nature, the modifier is associated to the electrode by adsorption, covalent binding, coating or even dispersion into a conductive matrix.

#### Minerals

Minerals such as silica-based materials, clays and zeolithes, are of particular interest for ion exchange voltammetry (IEV) (Wang, 1989). Basically, they act as an ion selective film inside which the analytical target is preconcentrated at open-circuit potential by an exchange process. In a second step, the analyte incorporated within the ion-exchanger film is detected by using an anodic stripping technique (Walcarius, 1998).

In France, the research on silica-modified electrodes with respect to heavy metals assay is mainly represented by the group of Walcarius. In 1997 this group published the first report dealing with silica-modified carbon paste electrode (SMCPE) devoted to electroanalysis, with Cu(II) as the analytical target (Walcarius and Bessiere, 1997). By using a 10 min preconcentration step and SWASV, a LOD of 2 nM was reached. This SMCPE exhibited a good reproducibility since up to 30 detection procedures were performed over a period of a week without any noticeable loss of sensitivity. However, this system suffered the classical drawback of CPE, namely a gradual dissolution process. In this particular example, another severe drawback was the necessary use of an ammonia medium in order to ensure Cu(II) accumulation via its interaction with surface silanolate groups. This pioneering work has been later extended to various silica-based materials and the influence of interfering species has been studied (Walcarius et al., 1999a). Clearly, this SMCPE failed at high ionic strength, since important cations concentration resulted in competition for the ion-exchange sites. A very similar study has been reported with Hg(II) as the analytical target (Walcarius et al., 2000). Using the same procedure and materials, Hg(II) has been found to suffer no influence of ionic strength and no particular interference with other metallic species, even Ag(I). This result has been explained taking into account the formation of soluble Hg(II) hydroxides in the experimental conditions used.

To overcome the drawback of adding a complexing agent in order to ensure metal accumulation, the group of Walcarius has developed several organically modified silica CPEs. Aminopropyl groups (Walcarius et al., 1999b; Etienne et al., 2001) or a carnosine dipeptide (Sayen et al., 2003) have been co-condensed with silane precursors to afford the desired functionalized silica materials. Cu(II) was detected with similar LODs to those reported in the former study (Walcarius and Bessiere, 1997) without adding anything to the accumulation medium. The aminopropyl-grafted silica CPE was successfully tested for Cu(II) detection in laboratory tap water (Etienne et al., 2001). Cu(II) suffered important competition from Co(II), Ag(I), and Hg(II) for the binding sites of the carnosine-modified silica CPE, thus limiting the practical usefulness of this sensor in real media (Sayen et al., 2003).

**Figure 4:**
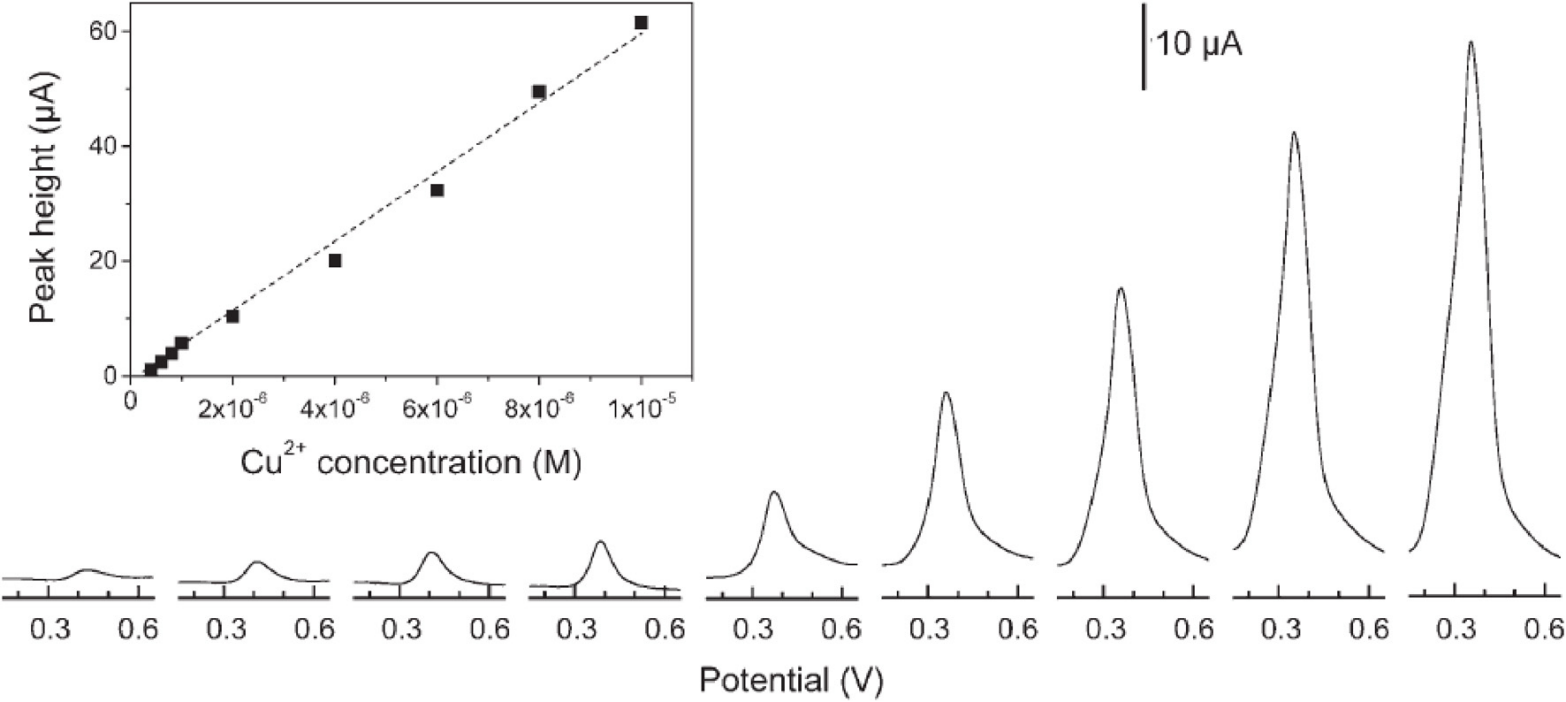
**Typical DPASV and calibration (inset panel) curves obtained for Cu^2+^ using a 10% amine functionalized silica film deposited on gold**. Reprinted with permission from Walcarius and Sibottier (2005). Copyright 2005 Wiley-VCH.

An interesting alternative to CPE has been proposed later by using silica films coated onto Au (Walcarius and Sibottier, 2005) or GC (Etienne et al., 2007; Sanchez and Walcarius, 2010) electrodes. Whereas these films have been prepared most of the time by a classical surfactant-templated synthesis (Etienne et al., 2007; Sanchez and Walcarius, 2010) an original electrochemically-induced sol-gel deposition has been also reported (Walcarius and Sibottier, 2005) (Figure 4). All these films were functionalized either by thiol or amine groups. Cu^2+^ (Walcarius and Sibottier, 2005), Ag^+^ (Etienne et al., 2007) and Hg(II) (Sanchez and Walcarius, 2010) were the analytical targets, and the LODs obtained were 40, 6, and 24 nM respectively.

From a more general point of view, all these studies on silica-based modifiers proved that the key feature with respect to analytical performances, and particularly sensitivity, is the analyte diffusion inside the porous structure of silica, thus making porosity a more predominant factor than the amount of surface silanol groups (Walcarius et al., 1998). Thus, mesoporous silica-based materials, which exhibit well-defined three-dimensional structures, appear to be much more adapted for heavy metal preconcentration than amorphous ones (Walcarius et al., 2003), whatever they are functionalized (Ganesan and Walcarius, 2008) or not (Walcarius et al., 1998). When functionalized, the amount of organic groups is also of importance, its effect on the analyte preconcentration passing by a maximum, since too much organic groups lead to a decrease in pore size which hampers the accessibility of the binding sites (Etienne et al., 2007; Ganesan and Walcarius, 2008; Sanchez and Walcarius, 2010).

Clays may also be used to perform IEV. These minerals exhibit relatively large specific surface areas and ion-exchange properties associated to an ability to sorb and intercalate many compounds. In a very close approach to what has been reported for silica-based material, their surface may be functionalized by organic groups (Navratilova and Kula, 2003), affording the possibility to tune charge selectivity of clays in IEV (Tonle et al., 2004).

In France, the group of Walcarius explored the potentialities of clays functionalized by organic groups and mixed with CPE toward Hg(II) (Tonle et al., 2003, 2005) and Pb(II) (Tonle et al., 2011) determination. With respect to Hg(II), the comparison of thiol-functionalized vs. amine-functionalized clays showed that the former one exhibited a better LOD (68 and 87 nM, respectively, using DPASV with 10 min accumulation), in accordance with the greater affinity of Hg for sulfur groups (Tonle et al., 2005). Thiol-functionalized clays have been also coated as thin films onto GC (Tchinda et al., 2007, 2009). The LOD has been greatly improved since a 6 nM value was reached using DPASV with only 3 min accumulation, the linear range being from 50 to 800 nM (Tchinda et al., 2007). It has to be noticed that increasing the accumulation time allowed another linear range to be observed from 4 to 20 nM together with a remarkable LOD of 0.5 nM (Tchinda et al., 2007).

#### Macrocyclic compounds

Macrocyclic compounds have received much attention in the last two decades due to their particular three-dimensional shape which provides a suitable cavity for selective complexation of heavy metals (Kolthoff, 1979). French groups particularly focused their research on cyclams (Goubert-Renaudin et al., 2009a,b; Nasraoui et al., 2010a,b), crown-ethers (Parat et al., 2006; Betelu et al., 2007) and calixarenes (Dernane et al., 2013; Rouis et al., 2013).

With respect to these latter compounds, Dernane et al. proposed to detect Cd^2+^ thanks to a *p*-tert-butylcalix[8]arene membrane deposited onto Au electrode (Dernane et al., 2013). In this work the metal cation coordination was favored by the presence of the four ketone groups on the calixarene, thus allowing a 0.1 μM LOD to be reached. However the results seemed a little bit cautious since detection was performed simply by CV without further preconcentration step. Moreover, the variation of peak current density as a function of the logarithm of metal concentration was proposed as a calibration plot, which makes non-sense with respect to classical equations of CV.

Rouis et al. have built an impedancemetric sensor dedicated to Hg^2+^ detection by immobilizing a β-ketoimine calix[4]arene derivative in a conducting poly(*p*-phenylene vinylene) membrane deposited onto indium-tin oxide (ITO) electrodes (Rouis et al., 2013). The main originality of this work was the study of light excitation effect of the β-ketoimine calix[4]arene while optimizing detection parameters. Charge transfer processes at the electrode/electrolyte interface were found to be improved under light excitation, thus providing an enhancement of the sensitivity toward Hg^2+^. The best results were obtained under blue light emission, providing a charge transfer resistance *R*_*ct*_ = 4.47 kΩ and a linear range from 0.1 nM to 0.5 μM.

**Figure 5:**
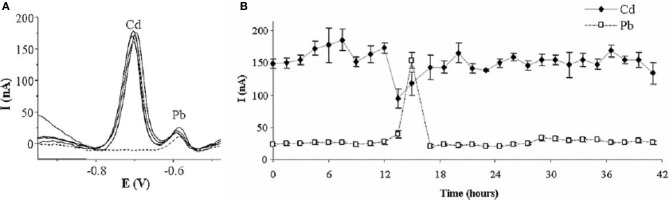
**(A)** SW Voltammogram recorded every 12 h for 42 h analysis by semicontinuous flow injection of tap water doped with Cd **(B)** Variation of Cd and Pb peak currents over the 42 h. Reprinted with permission from Betelu et al. (2007). Copyright 2007 Wiley-VCH.

Parat et al. reported the use of a thin-film mercury-coated screen-printed carbon electrode covered by a crown-ether based membrane for the determination of Cd and Pb (Parat et al., 2006). The crown-ether membrane aimed at protecting the active surface from interferences during the analysis. The size of crown-ethers cavity has been proved to impact the performances of the electrode. The best results have been obtained for the smallest crown-ether, because its cavity size fitted well to metal ions radii, although Pb was a bit favored. LODs of 6 nM and 8 nM for Cd^2+^ and Pb^2+^, respectively, have been reached by using LSASV with 2 min preconcentration. Tests were successfully conducted on river water and soil solution extracts. This system was then applied in a later work to the semi-continuous monitoring of Cd and Pb in tap water by FIA and afforded reproducible results for 42 h (Betelu et al., 2007) (Figure 5).

Goubert-Renaudin et al. have developed a new family of functionalized cyclams they grafted onto silica materials and mixed to CPE (Goubert-Renaudin et al., 2009a,b). In the first work dedicated to Cu(II) detection, the functionalization aimed at stabilizing cyclams on silica in order to improve the sensor lifetime. However, increasing the number of functionalizations per cyclam lead to an increase in cycle rigidity, which has been correlated to lower performances with respect to Cu(II) uptake (Goubert-Renaudin et al., 2009a). The modified CPE afforded good stability, with a 7% relative standard deviation for 45 measurements and was successfully tested on tap water. In a second work, dealing with Pb(II) determination, the aim of the functionalization was to improve the analyte preconcentration step by favoring complexation (Goubert-Renaudin et al., 2009b). Thus, the inverse trend was observed, i.e., the more functionalized the cyclam, the better the performances. The best system allowed LODs down to 2.7 nM to be reached. Among potential interfering species, only Hg(II), Cd(II), and Cu(II) gave rise to significant loss of signal, mainly because of competition for the binding sites. The group of Geneste also used such kind of cyclams to functionalize graphite felt electrodes (Nasraoui et al., 2010a,b). Accumulation of Pb(II) at open-circuit potential by flowing the aqueous solution to analyze, followed by LSASV allowed a 25 nM LOD to be reached. However, this system exhibited relatively poor stability since a 20% decrease of the response was reported after regeneration.

#### Polymers

Electroactive surface modification by means of polymer deposition or electrodeposition represents a broad research field, leading to numerous papers every year. With respect to trace metals analysis, polymer films allow the immobilization on the electrode of a large number of ligands which may complex metal ions to be accumulated (Trojanowicz, 2003; El Kaoutit, 2012; Li et al., 2012). The polymers used for surface modification may be natural or prepared purposely by chemical synthesis. However, with respect to French groups' research activities, no work was found dealing with natural polymers dedicated to trace metal detection. The only papers found in the literature were about polysaccharides (Crini, 2005) and chitosan (Vieira et al., 2011) and concerned heavy metals removal.

**Figure 6:**
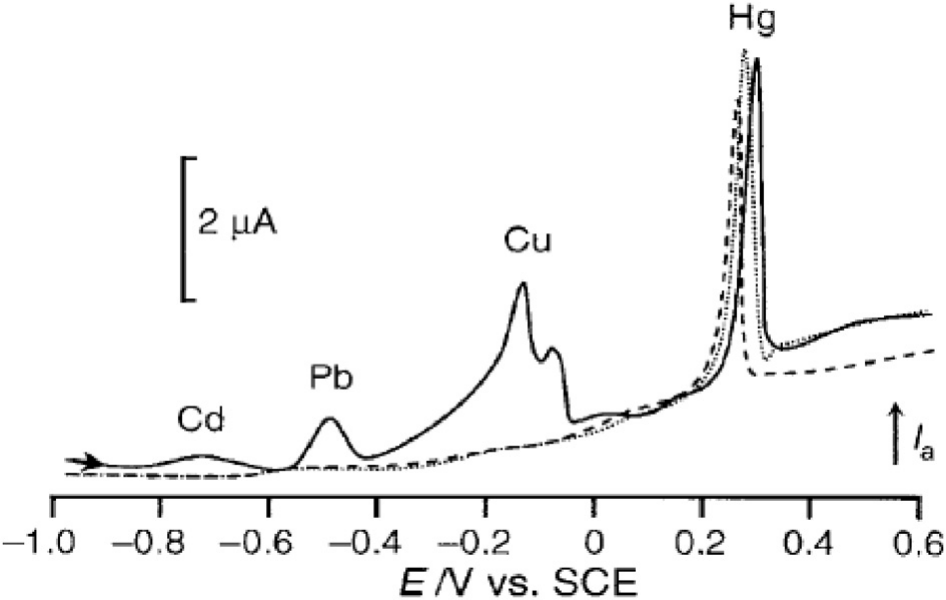
**DPV curves recorded at a poly(EDTA-like) film modified carbon electrode in acetate buffer containing Hg(OAc)_2_ (dotted line), Cu(OAc)_2_, Cd(NO_3_)_2_, Pb(NO_3_)_2_, Hg(OAc)_2_ (full line)**. Reprinted with permission from Buica et al. (2009b). Copyright 2009 Wiley-VCH.

Concerning chemically synthesized polymers, the group of Moutet has developed two different groups of substituted polypyrrole derivatives for trace metal determination. The first one is based on “poly(pyrrole-EDTA like)” and takes advantage of the well-known complexing properties of EDTA to improve metal preconcentration (Heitzmann et al., 2007; Buica et al., 2009a,b). These studies were devoted to the assay of Cu(II), Pb(II), Cd(II), and Hg(II) (Figure 6). In the first two ones, the selectivity of the modified electrode has been tuned by varying the accumulation time and the pre-structuration of the polymer. The film thickness was also proved to influence the selectivity. However, the electrode was insensitive to Cd(II) whatever the conditions adopted. To overcome this problem, Heitzmann et al. have chosen an imprinted polymer strategy: the polymer was electrodeposited in the presence of Cd(II) ions which were then removed from the metallopolymer film (Heitzmann et al., 2007). The resulting functionalized electrode was thus able to detect Cd(II) as well as the other three metal cations. By introducing 4 pyrrole fragments on the same EDTA skeleton instead of only two in the former studies, an enhanced stability and a better controlled dimensionality was conferred to the polymer, thus making the sensor response independent on the film thickness (Buica et al., 2009a). The global complexing capability of the polymer was also greatly improved by the presence of 2 amine and 4 amide coordinating groups per monomer unit. The second group of polymers developed by the group of Moutet is based on poly(pyrrole-malonic acid) (Heitzmann et al., 2005; Pereira et al., 2011). Here, the analyte complexation occurred with the anionic form of malonic acid, which is known to easily coordinate various metal ions. This sensor was also tested with Cu(II), Pb(II), Cd(II), and Hg(II). It allowed LODs around 10^−10^ M to be reached for each metal cation, and exhibited good stability since the same current response was obtained for 2 assays at a 3 weeks interval using the same electrode stored without any particular precaution.

Another kind of polymer based on styrene units gave rise to one report by Moutet (Rivas et al., 2006). Styrene was copolymerized with acetamide acrylic acid or itaconic acid. These latter are hydrophilic whereas styrene is hydrophobic, thus providing interesting mixed properties to the resulting copolymers. The incorporated carboxilate groups were used for the accumulation and detection of Pb^2+^ but the system exhibited poor performances since the response was linear only in the narrow range from 10^−5^ to 10^−3^ M.

Bessbousse et al. proposed a more sophisticated system based on a nanoporous β-poly(vinylidene fluoride) (β-PVDF) membrane (Bessbousse et al., 2011). The nanopores were further functionalized by track-etched poly(acrylic acid) (PAA), and thin porous Au films were sputtered on each side of the membrane. This very sophisticated electrode has been proved to detect Pb^2+^ but no very clear analytical results were provided.

Polythiophene also gave rise to one report (Zejli et al., 2007). The polymer was electrodeposited on a Pt electrode by cyclic voltammety and used to detect Ag(I) by DPASV taking advantage of the inductive effect of the C-S dipole of thiophene units. However, the linear range was found to be very narrow, from 0.65 to 9.3 μM.

A contribution from the group of Noguet has also to be noticed (Yasri et al., 2011). In this original work, a graphite electrode coated by a 3,4-poly(ethylenedioxythiophene):poly(styrene sulfonate) [PEDOT:poly(styrene sulfonate)] copolymer was used to detect Pb^2+^ by chronoamperometry at −0.35 V. The linearity range was from 2 to 100 nM and the LOD was 0.19 nM for 30 s accumulation at −0.65 V. The system exhibited good stability since only a slight decrease was noticed after 11 days. It was also successfully tested for the determination of Pb^2+^ in different vegetables extracts.

In order to improve the detection limit the group of Chevalet exploited the high resolution power of a multi-pulse electroanalytical method, namely multiple square wave voltammetry (MSWV) (Fatouros et al., 1986; Krulic et al., 1990). MSWV is based on the superimposition of several pairs of opposite pulses of constant amplitude on each step of a staircase waveform. However MSWV differs from previously described DAPV by the electrode response: instead of the double cathodic and anodic current recorded in the latter case, MSWV response results in the integration of the successive currents. The MSWV-DD (DD for double differential) technique was used in combination with a Nafion-coated electrode for the determination of trace species like methylmercury (Moretto et al., 1999) and Fe (Ugo et al., 2001). The perfluorinated cation exchanger Nafion was used to preconcentrate the analyte and was simply deposited on a GC disk by droplet evaporation. The detection capabilities of this polymer-coated electrode combined to the sensitive MSWV-DD method allowed a calculated LOD down to 45 pM to be reached for methylmercury (Moretto et al., 1999).

#### Nano-scaled materials

During the last two decades, nano-scaled materials have aroused a great interest with respect to analytical applications due to their specific physico-chemical properties (Murray, 2008). Improvements resulting from those nanomaterials for electroanalysis are manifold: enhanced diffusion of electroactive species, higher effective surface area of nanoparticles (NPs), electrocatalytic and conductive properties, improved selectivity and higher signal-to-noise ratio. With respect to trace metal analysis, gold nanoparticles (AuNPs) are the most commonly used material (Lin et al., 2011; Liu et al., 2011). They can be obtained either by chemical or electrochemical ways.

The French contribution to this topic is very recent, and the corresponding works all considered the structuration of the nanoparticle-based modified electrode to be a key feature with respect to analytical performances.

Ottakam Thotiyl et al. designed a multilayer arrangement of citrate-capped AuNPs immobilized by a thiol group onto a Au electrode for the detection of As(III) (Ottakam Thotiyl et al., 2012). The anionic AuNPs were deposited layer-by-layer alternatively with cationic polyelectrolyte to afford a layered nanocomposite film. As(III) was detected by its electrocatalytic oxidation using DPV without any accumulation step, and a LOD of 0.48 μM was reported. This study evidenced a strong correlation between the amperometric response and the number of layers of the nanocomposite film.

**Figure 7:**
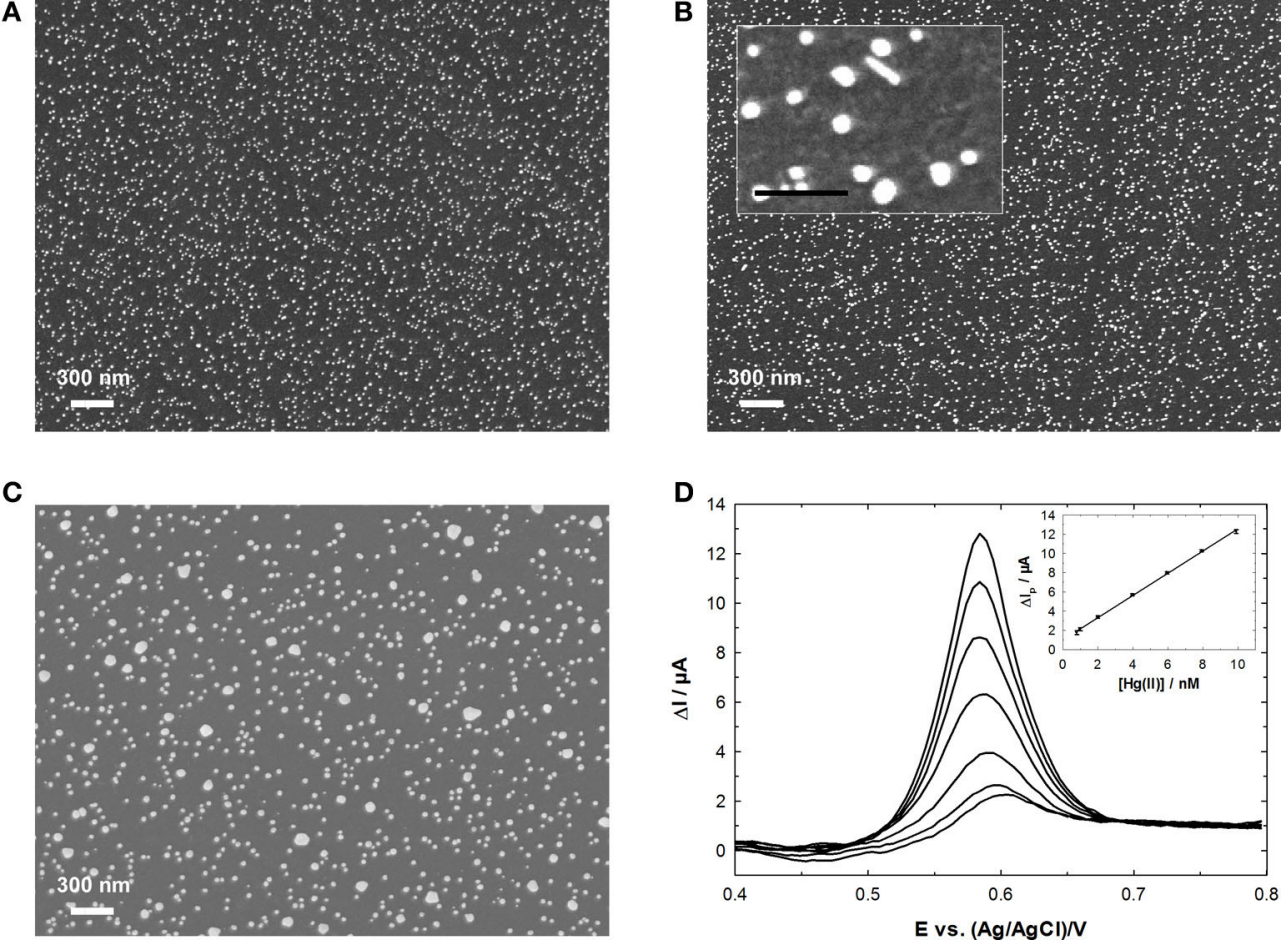
**AuNPs electrodeposited onto GC from a 0.25 mM HAuCl_4_ solution using: (A) chronoamperometry; (B) potentiostatic double pulse; (C) cyclic voltammetry**. **(D)** SWASV response and calibration curve obtained in the Hg(II) concentration range 0.8–9.9 nM using electrode **(A)**. Adapted from our own results published in Hezard et al. (2012b), Copyright 2012 Elsevier.

Our group has developed a Hg(II) sensor based on GC electrode functionalized by electrodeposited AuNPs and has studied the influence of the electrodeposition method on the analytical performances (Hezard et al., 2012a,b). Namely, cyclic voltammetry, chronoamperometry and potentiostatic double-pulse were used. It was shown that both the electrodeposition mode and the charge used for the Au precursor reduction had a dramatic influence on the size and density of AuNPs (Figure 7). These latter two parameters were strongly correlated to the analytical performances: the best results were obtained for dense deposits of small NPs (Hezard et al., 2012b). By using a 5 min accumulation time and SWASV, a LOD of 0.4 nM was reached.

An important development in the frame of nano-scaled materials concerns single (SWCNTs) or multi-walled carbon nanotubes (MWCNTs) since their discovery in 1991 (Ijima, 1991). Their unique structure offers very interesting properties such as high specific surface area, high chemical stability, good electrical conductivity and adsorption capacity, which give rise to wide applications in electronics, composite materials, energy storage, and of course sensors (Fam et al., 2011). With respect to this latter domain the association of various CNT functionalization procedures, including organic (polymers, proteins…) or inorganic (metal nanoparticles, metal oxides…) modifiers and several transducing modes allowed the elaboration and development of chemical, gas, humidity, biomedical or environmental sensors which have been the subject of several reviews (Jacobs et al., 2010; Vashist et al., 2011; Gao et al., 2012). Extensive international researches have been made to assay heavy metals by using unmodified (Yue et al., 2012) or CNTs modified either with cysteine (Morton et al., 2009), thiacalixarene (Wang et al., 2012), Sb nanoparticles (Ashrafi et al., 2014) or with imprinted polymeric nanobeads (Rajabi et al., 2013), most of the time associated with ASV. They allowed Pb, Hg, Cu, Cd, or Zn to be detected down to 3 nM and in linear concentration ranges up to 7 μM. Surprisingly, to the best of our knowledge, no French research team has exploited CNTs for heavy metals detection yet. Interest has mainly focused on micro and supercapacitors (Ghimbeu et al., 2011), batteries (Carn et al., 2013) and energy storage devices (Sathiya et al., 2011) or biofuel cells (Both Engel et al., 2013; De Poulpiquet et al., 2013). Concerning sensors works essentially dealt with gaz sensors (Bondavalli, 2010) and above all enzymatic (Singh et al., 2013), RNA (Tran et al., 2013) and DNA (Zhang et al., 2011)-based electrochemical biosensors.

#### Biosensors

In general terms, a biosensor is defined as an analytical tool associating a bioactive compound (mono- or multi-enzyme system, antibody, microorganism) which can specifically recognize species of interest and a transducing element (Frew and Hill, 1987). Thus, it may be viewed as a particular kind of CME in which the modifier is a biological element. Enzymes are known as good modifiers with respect to heavy metals since these latter often strongly inhibit enzymatic reactions (Dennison and Turner, 1995).

The group of Chovelon reported several works about conductometric biosensors based on *Chlorella vulgaris*, a microalgae the cell walls of which bear enzymes such as alkaline phosphatases and esterases (Chouteau et al., 2004, 2005). In both papers, the microorganism was immobilized onto interdigitated Pt electrodes using bovine serum albumin and glutaraldehyde as a crosslinker. In the first work, Cd^2+^ was detected as low as 8.9 nM but at least one hour was needed to obtain such a result. The second study (Chouteau et al., 2005) provided several improvements since both Cd^2+^ and Zn^2+^ were detected with a 89 nM and 0.15 μM LOD, respectively, but with a significantly lower exposure time (ca. 30 min). It has to be noticed that bioassays were found to reach a similar LOD to the biosensor but only after 4 h exposure time, illustrating the wise enzyme immobilization strategy used. Finally, an originality of this latter work was that the biosensor was also capable to detect pesticides, taking advantage of the fact that esterases were selectively inhibited by these organic compounds whereas alkaline phosphatases were not affected.

Tekaya et al. have also reported a conductometric biosensor based on alkaline phosphatase from the cyanobacterium *Arthrospira platensis* (Tekaya et al., 2013) deposited on the ceramic part of interdigitated Au electrodes. In this case, inhibition measurements were performed after 24 h incubation, allowing LOD down to 10^−20^ M to be reached for both analytical targets, namely Cd^2+^ and Hg^2+^. Despite this very appreciable LOD, no information was provided about sensor lifetime. This is mainly due to the fact that the authors considered their study as a proof of concept aiming at providing a global response to the presence of heavy metals.

A last conductometric biosensor was reported 2 years ago by Soldatkin et al. (2012). This sensor consisted in a three-enzyme system prepared by mixing invertase, mutarotase and glucose oxidase with bovine serum albumin. Although a bit complicated, the sensor exhibited good sensitivities toward Hg^2+^ and Ag^+^ without experiencing any interference from organic compounds. After a relatively short incubation time (ca. 10–20 min), the LOD were found to be 25 and 100 nM for Hg^2+^ and Ag^+^, respectively. It has to be noticed that this biosensor was very selective since Cd^2+^, Zn^2+^, Ni^2+^, Pb^2+^, Cu^2+^, and Co^2+^ did not affect the enzymes activities. Interestingly, the authors have investigated a reactivation procedure: dipping the biosensor into EDTA or cysteine solution for 45 min allowed a significant reactivation of the enzymes together with the identification of the inhibitor metal, since EDTA reactivated Ag^+^ inhibition only whereas cysteine was selective to Hg^2+^ inhibition.

The group of Cosnier proposed an amperometric sensor toward Hg(II) based on the same three enzymes which were entrapped in a clay matrix deposited on Pt electrode (Mohammadi et al., 2005). The electrochemical response was provided by the oxidation of the enzymatically produced hydrogen peroxide. After 20 min incubation, Hg(II) was detected in the range 10 nM to 1 μM. It has to be noticed that several Hg species have been tested, such as methylmercury or phenylmercury, all of them leading to invertase inhibition. Depending of the analytical target, the biosensor recovery by using cysteine was more or less effective, but never complete. The system was quite selective to Hg(II) since all metal cations tested induced no interferences except Ag^+^.

Finally, the group of Burstein has reported 20 years ago a bi-enzymatic system coupled to an oxygen sensor (a Clark electrode) which offered an original solution to the enzyme recovery issue (Gayet et al., 1993). In this work, several enzyme combinations were tested but the most interesting one was that involving L-lactate oxidase and L-lactate dehydrogenase. Actually, the former enzyme was immobilized on the membrane of the oxygen sensor whereas the latter one remained in solution. Since only L-lactate dehydrogenase was affected by the presence of heavy metal cations, the sensor regeneration simply consisted in a renewal of the solution containing this very cheap enzyme. Unfortunately, the interest of this system was limited due to the relatively poor LOD obtained for the different analytical targets.

### The particular issue of trace metal speciation

In addition to the monitoring of total heavy metals concentrations in the environment, speciation analysis provides very useful complementary informations. However speciation analysis often implies the determination of very low concentrations of minor species, typically in the range of nM to pM or even lower. Hence electrochemistry and especially anodic stripping techniques are particularly well adapted to such kind of analysis.

For instance, Bourgeault et al. studied dissolved Cu speciation and bioavailability in freshwaters by using DPASV, diffusive gradient in thin films and ISE, and compared their results to those obtained by modeling (Bourgeault et al., 2013). It was found that Cu accumulation in aquatic mosses was better correlated to the weakly complexed Cu species measured using DPASV than to free Cu concentration measured using an ISE, thus highlighting the contribution of electrochemical techniques to speciation studies.

In a close approach combining DPASV and conductometry, Terbouche et al. examined the complexation of Zn(II) and Cd(II) by new humic acids from Algeria, these latter class of natural polymers being known to have an influence on heavy metals biogeochemical cycle and bioavailability (Terbouche et al., 2011). Based on the strong complexing capacities evidenced in this work, the authors suggested their humic acids to be used for metal uptake.

A last speciation study combining electrochemical techniques was provided very recently by Rotureau (2014). Here the already discussed “AGNES” technique was used for the determination of free metal in solution while SCP at scanned deposition potential (SSCP) allowed dynamic speciation information to be reached. This latter technique consists in performing classical SCP with varying deposition potentials, and then plotting the transition time as a function of the deposition potential (van Leeuven and Town, 2002). Rotureau's work dealt with Cd and Pb speciation dynamics in clay minerals, and it was shown that the interaction of Cd with clays may be described as a chemically homogeneous, labile system over a wide pH range whereas strong pH effects were evidenced in the case of Pb.

SSCP has also been used by Parat et al. to study Cd speciation parameters in the presence of several ligands, namely nitrilotriacetic acid (NTA) and 2,6-pyridinedicarboxylic acid (PDCA) (Parat et al., 2011b). By using a Hg film SPE and a 60 s deposition time, a LOD of 2.2 nM was reached and some information upon speciation extracted. In particular, it was demonstrated that Cd-NTA complex is less labile than Cd-PDCA.

It has to be noticed that classical SCP has been used by the group of Riso for the determination of Se(IV) and total Se in seawater on a HgFE (Riso et al., 2004). However in this case, speciation information was reached by tuning the experimental parameters of water pretreatment, the electrochemical ones remaining nearly the same. Thus, Se(IV) concentration was determined by performing SCP on untreated water, whereas total Se was reached by analyzing UV-digested water.

Among all anodic stripping methods, pseudopolarography is particularly well-suited to provide useful information on metal speciation in model solutions and in natural water. This electrochemical technique allows thermodynamic and kinetic properties of the different forms of metal ions to be reached. The current-potential curves obtained, called pseudopolarograms, depict the influence of the metal deposition potential on the peak height, the peak potential or the half-peak width of the subsequent anodic voltammogram. The shifts in peak potentials as well as the changes in the slope of the curve indicate a modification in the reaction kinetics or reversibility. These evolutions make it possible to discriminate between different fractions of metals, including free or labile species and electroinactive metal complexes, and to detect the presence of complexing ligands at low concentration. Compared to classical linear or pulsed voltammetry, pseudopolarography allows the speciation of trace metals at natural concentration level (generally less than nM). Pizeta et al. have taken benefit of this properties to detect Pb(II) in sediment and interstitial water with an iridium solid microelectrode covered by a thin mercury film (Pizeta et al., 2005). The electrode sensitivity varied between 4 and 20 nA /μM and its LOD was about 30 nM. The amperometric response was repeatable enough, avoiding memory effect and electrode surface blocking. Particularly the results highlighted the existence of strong complexes in sediment and in non-filtered interstitial water with high pH. More recently this procedure was successfully applied for the study of the interactions of natural (NOM) or dissolved (DOM) organic matter with Cu and Zn (Louis et al., 2008; Nicolau et al., 2008) in seawater sample. The unpolluted marine water containing DOM about 1 mg L^−1^, measurements were possible in the latter case after concentrating DOM in large water volumes by nanofiltration and reverse-osmosis. Anyway the results gave comprehensive elements on the role of marine DOM in metals speciation: the dependence of pseudopolarograms with pH clearly indicated the differences of DOM sites for Cu and Zn complexation regarding their stability and acidity constants. The main drawback is the time-consuming of the method which requires a set of anodic stripping voltammograms to be recorded for each pseudopolarogram.

To end this section dedicated to heavy metal speciation, let's talk about some works which are certainly not the most original ones in terms of electroanalytical method, but present particularly interesting automated systems. Zaouak et al. recently developed a system for Cd semicontinuous monitoring which allowed the determination of total and bioavailable Cd (Zaouak et al., 2010a,b). The analyses were performed using a Hg film deposited on a SPE, this latter electrode being integrated in a fully-automated device allowing all the operations needed for a complete set of measurements. Thus, a measurement cell, a storage cell and a plating cell for the electrode renewal were comprised in the whole device, together with a hydraulic conveyor which allowed the three-electrode system to move from one cell to another. This automated device was tested on tap water by recording measurements every 160 min for 18.5 h and exhibited a LOD of 1.78 nM. Total and bioavailable Cd were obtained by adjusting the pH value of the treated water.

Another example of automated electroanalytical device was reported by Hezard et al. for Fe(III) and Fe(II) quantification in acid mining drainage water (Hezard et al., 2009). In this particular case, the redox species were concentrated enough to allow direct determination using an unmodified Pt electrode. Moreover, the presence of sulfide anion S^2−^ in acid drainage water induced an important negative shift in potential for O_2_ reduction, resulting in a well-separated reduction wave for Fe(III). Thus, both Fe(III) and Fe(II) were determined using a single potential scan without deaerating the solution. An originality of this work was that both sample renewal and hydrodynamic control were achieved by a fluidic system based on gravity instead of a pump.

## Conclusion

From an historical point of view, the unique properties of the conventional mercury electrode have been at the origin of considerable efforts with respect to electrochemical methods devoted to heavy metals detection in water. Nowadays the great experience in polarographic techniques coupled with the improvements in data processing for the monitoring of pulsed potential sequences is still encouraging several groups to exploit the background on this electrode. Nevertheless recent trends have focused on mercury-free electrode since this metal will be totally prohibited in the next few years (European Directive 2008/105/EC). Depending on whether the goal is heavy metal pollution detection in industrial waste or drinking water analysis, detection limits in the μM or nM have to be reached, respectively. In the former case ISEs are well-suited due to the simplicity of the potentiometric measurement and the good understanding of the sensing mechanism. On the other hand the detection of trace metals requires more sensitive methods. In that way CMEs represent a good alternative toward expensive, inconvenient analytical methods developed in laboratory. The combination of amperometric transduction with the specificity induced by the modifier makes it possible to propose reagentless sensors exhibiting high sensitivity and good selectivity. Furthermore the large range of available strategies for surface modification allows multielectrodes or micro array sensors to be designed for simultaneous multicomponent analysis in complex media. Finally a new generation of analytical devices called “micro total analysis system” (μTAS) is being developed for the “on line” monitoring of real samples. These devices integrate not only the sensing element but also some complementary actuators (pumps, valves, microfluidic channels) to obtain an all-integrated automatic monitoring system which includes a calibration procedure. A few prototypes have been recently proposed by using microtechnology manufacturing and semi-conductor processing (Jothimuthu et al., 2011; Jung et al., 2011) but are still at the emerging state. Still a good challenge for French research community!!

### Conflict of interest statement

The authors declare that the research was conducted in the absence of any commercial or financial relationships that could be construed as a potential conflict of interest.

## Abbreviations

ASV: anodic stripping voltammetry
BDD: boron doped diamond
BiFE: bismuth film electrode
CME: chemically modified electrode
CPE: carbon paste electrode
CSV: cathodic stripping voltammetry
CV: cyclic voltammetry
DAPV: differential alternative pulses voltammetry
DLC: diamond like carbon
DME: dropping mercury electrode
DPASV: differential pulse anodic stripping voltammetry
DPV: differential pulse voltammetry
FIA: flow injection analysis
GC: glassy carbon
HgFE: Hg film electrode
HMDE: hanging mercury drop electrode
ICP-MS: inductively coupled plasma mass spectroscopy
IEV: ion exchange voltammetry
ISE: ion selective electrode
LOD: limit of detection
LOQ: limit of quantification
LSASV: linear sweep anodic stripping voltammetry
LSSV: linear sweep stripping voltammetry
LSV: linear sweep voltammetry
MSWV: multiple square wave voltammetry
MWCNT: multi-walled carbon nanotube
NPV: normal pulse voltammetry
NPs: nanoparticles
SCP: stripping chronopotentiometry
SMCPE: silica-modified carbon paste electrode
SMDE: static mercury drop electrode
SPE: screen printed electrode
SSCP: stripping chronopotentiometry at scanned deposition potential
SWASV: square wave anodic stripping voltammetry
SWCNT: single-walled carbon nanotube
SWCSV: square wave cathodic stripping voltammetry
SWV: square wave voltammetry
TFME: thin film mercury electrodes
WFD: water frame directive.
